# Pathology

**Published:** 1879-08

**Authors:** 


### PATHOLOGY.

HERVIEUX AND PASTEUR ON SEPTICÆMIA. The following abstract of a discussion, in the Academy of Medicine, in Paris, session of May 6th, 1879, between the distinguished author Dr. Hervieux and M. Pasteur, on the subject of the "germ theory" and puerperal septicæmia, is reported in *Le Progrés Medical*, May 10, 1879.

"In reply to a previous communication by M. Pasteur on puerperal septicæmia, M. Hervieux called attention to the obscurity which surrounds the doctrine of that illustrious experimenter. Is the supposed microbion of puerperal septicæmia the product or the productive cause itself of the malady? If the pathogenetic power is the attribute of the microbion, that parasite should be discovered only where puerperal septicæmia exists. Now, according to Pasteur, the microbion is found everywhere, in common water. On the other hand, all lying-in women who use water, either as a drink, or in injections, or for purposes of cleanliness, should be invaded by this infectious parasite. The mortality of these patients is too variable a factor to be reconciled with any such hypothesis. This great question of the almost exclusive predilection of puerperal septicæmia for certain localities — notably for the obstetrical wards of a hospital — can be resolved by a single word: *agglomeration*.

"How and why does the supposed microbion of puerperal septicæmia appear with its septic characteristics in a lying-in hospital? The organisms discovered by Pasteur in the blood and lochial discharges of two women who have succumbed to puerperal septicæmia do not prove that they were the productive cause of puerperal intoxication.

The force which we invoke, and which surpasses the energy of the so-called septic parasites, is a special poison engendered by crowding together the newly delivered mothers. The microbion is not the poison; it is the parasite of the poisoned tissue; it is as truly a parasite as thrush in the mouth of a patient with enteritis, or as the lice upon the scalps of young children afflicted with eczema capitis. The germ theory will never become really fruitful until clinical observation shall be in complete accord with physiological experiment. As for the application of the germ theory to puerperal septicæmia, M. Hervieux considers our observations of the hypothetical microbion of puerperal septicæmia as so many elements out of proportion with the fundamental facts of nosology. The microbion, reputed to be so fatal to the population of pregnant females, must be an ordinarily inoffensive parasite, for it is found everywhere, and can be very easily detected in water. It is only — according to the hypothesis — in certain conditions that it acquires the septic character, particularly when associated with the microbion which Pasteur considers as the generator of pus. It must, therefore, be a creature sometimes single, sometimes double, sometimes inoffensive and sometimes murderous in its character. It cannot, then, be like all other virulent agents, always identical with itself.

M. Pasteur expressed regret that he could not reply with facts of a positive, unquestionable and decisive nature, and declared that he had never announced the existence of a parasite peculiar to puerperal septicæmia (!) However, the facts which he had published appeared to him very encouraging and capable of explaining hypotheses which would probably at some future time become certainties.

M. Hervieux declared his willingness to agree with M. Pasteur from the moment of his acknowledgment of the non-existence of the puerperal microbion, provided only that he will busy himself with actual facts. " As for M. Pasteur's charge that I do not believe in the germ theory, I will reply that if such were not the case, I would not suffer myself to discuss the application of this theory to puerperal septicæmia, which would lead me to a blind application of the treatment which is its logical consequence."

